# Editorial: Bioinformatics of Non-Coding RNAs with Applications to Biomedicine: Recent Advances and Open Challenges

**DOI:** 10.3389/fbioe.2015.00156

**Published:** 2015-10-08

**Authors:** Alessandro Laganà, Alfredo Ferro, Carlo Maria Croce

**Affiliations:** ^1^Department of Genetics and Genomic Sciences, Icahn School of Medicine at Mount Sinai, New York, NY, USA; ^2^Department of Clinical and Molecular Biomedicine, University of Catania, Catania, Italy; ^3^Department of Molecular Virology, Immunology and Medical Genetics, Comprehensive Cancer Center, The Ohio State University, Columbus, OH, USA

**Keywords:** ncRNA, miRNA, lncRNA, RNA editing, NGS, biomedicine

The recent advances in the functional characterization of non-protein-coding RNAs (ncRNAs) have represented a major breakthrough in the life sciences. Large-scale projects, such as ENCODE, have shown that the human genome is pervasively transcribed and that a large proportion of the mammalian transcriptome consists of ncRNA transcripts. ncRNA genes can be roughly classified into short ncRNAs (<200 nt) and long ncRNAs (>200 nt).

The first class includes well characterized, infrastructural molecules, such as rRNA, tRNA, and snoRNA, which have a housekeeping role in essential processes like splicing and translation, and regulatory ncRNA such as miRNA and piRNA, which are involved in post-transcriptional regulation of gene expression and in the silencing of transposable elements during germ line development, respectively.

The second class of ncRNA consists of longer transcripts that are still poorly characterized mostly due to their heterogeneity in size, structure, and biogenesis. LncRNA genes outnumber short ncRNAs and are probably more abundant than protein coding genes. Such RNAs exhibit various degrees of conservation and are often polyadenylated and tissue-specific. Accumulating evidence indicates that they likely have a broad range of functions, including chromatin remodeling, gene regulation, and protein transport and trafficking.

Genetic and epigenetic aberrations affecting ncRNA gene sequences and their expression have been linked to a variety of pathological conditions, including cancer, cardiovascular, and neurological diseases.

Recently, high-throughput sequencing techniques have enabled the study of entire transcriptomes at single nucleotide resolution, providing unprecedented details of their organization, expression, modifications, and structure. Bioinformatics tools constitute an essential resource for ncRNA research, providing a powerful means to organize, integrate, and analyze the huge amount of data generated by such technologies.

The aim of this Research Topic is to review current knowledge, introduce novel methods, and discuss open challenges of this exciting and innovative field in connection with the most important biomedical applications. We have collected five original research and methods articles and four reviews, spanning the full scope of the Research Topic.

Two excellent reviews focus on the discovery of ncRNA from NGS data. Kang and Friedländer (2015) surveyed computational tools to predict animal miRNAs from short RNA sequencing data (RNAseq). The authors covered the basics of miRNA prediction, reviewed several methods, described the algorithms, and discussed their strengths. They also described algorithms for specific cases, such as prediction from massively pooled data or in species without reference genomes, and discussed challenges and future directions of the field. Veneziano et al. (2015), instead, provided a more general state-of-the-art coverage of the computational approaches for the discovery and analysis of small and long ncRNA through NGS techniques.

The detection of miRNAs from NGS data becomes an even more challenging task when sequence variants, termed isomiRs, are taken into account. IsomiRs were initially considered sequencing artifacts, but evidence showed that they are functional variants with a specific biological role. Muller et al. (2014) introduced IsomiRage, a streamlined pipeline to identify and analyze isomiRs from next generation sequencing data. The tool is able to distinguish canonical miRNAs from templated and non-templated isomiRs, including 5′- and 3′-extended and trimmed variants.

Two articles of this collection concern RNA editing, a dynamic, widespread process that alters the sequence of RNA transcripts. In particular, A-to-I editing is the most common RNA post-transcriptional modification in human and involves the deamination of adenosine (A) to inosine (I), which is recognized as guanosine (G) by all cellular machineries. Editing may alter both coding and non-coding sequences, with important functional consequences. Nigita et al. (2015) presented a comprehensive state-of-the-art review of databases and computational approaches for the discovery and the analysis of RNA editing, with particular emphasis on ncRNA. They summarized current knowledge and discussed potential consequences of RNA editing on ncRNA, pointing out the lack of tools specifically designed for the detection of editing alterations in lncRNA sequences. This gap was actually addressed by a methods article in our collection by Picardi et al. (2014). They described a novel computational approach to reliably detect A-to-I editing events in human lncRNAs through NGS, based on their previously published package called REDItools. In the presented article, the authors showed the potential of their tools in recovering A-to-I candidates from RNAseq data and provided guidelines to improve RNA editing detection in non-coding RNAs, with specific focus on lncRNAs.

This collection also includes three articles concerning data integration and functional analysis. In the first one, Bonnici et al. (2014) introduced ncRNA–DB, a novel database of ncRNA interaction in human. The database integrates associations among ncRNA, protein coding genes and diseases. It can be searched by a web-based or a command line interface and is also accessible through a Cytoscape app called ncINetView. The second paper, by Alaimo et al. (2014), described ncPred, a novel tool that predicts ncRNA-disease association through tripartite network-based inference. The results of the experimental analysis show that the tool is able to predict more biologically significant associations than its competitors. In the third paper, Li et al. (2015) performed a large-scale integration of publicly available RNA binding protein (RBP) binding sites generated by high-throughput CLIP-Seq technology and identified thousands of RBP–lncRNA interactions. The authors reported combinatorial effects among RBPs and discovered hundreds of disease-related SNPs in RBP binding sites in lncRNA.

The collection also includes a review of design principles and computational tools for the design of synthetic RNAs for gene regulation (Lagana et al., 2014). The article provided guidelines for the design of siRNA, artificial miRNA, antagomiRs, miRNA sponges, and small guide RNA for CRISPRi, and presented strengths and limitations of the different technologies.

Bioinformatics of ncRNA is a vast and rich field and the papers that we selected for this Research Topic address some of its most exciting and pressing challenges. We believe that this volume represents a valuable and useful resource and hope it will be of interest to the many researchers involved in ncRNA research.

## Conflict of Interest Statement

The authors declare that the research was conducted in the absence of any commercial or financial relationships that could be construed as a potential conflict of interest.
